# Epidemiologic Features of NSCLC Gene Alterations in Hispanic Patients from Puerto Rico

**DOI:** 10.3390/cancers12123492

**Published:** 2020-11-24

**Authors:** Ruifang Zheng, Zhiwei Yin, Albert Alhatem, Derek Lyle, Bei You, Andrew S. Jiang, Dongfang Liu, Zsolt Jobbagy, Qing Wang, Seena Aisner, Jie-Gen Jiang

**Affiliations:** 1 Department of Pathology, Immunology & Laboratory Medicine, Rutgers New Jersey Medicine School, Newark, NJ 07103, USA; zhengru@njms.rutgers.edu (R.Z.); yinz@mskcc.org (Z.Y.); aa1919@njms.rutgers.edu (A.A.); yoube@njms.rutgers.edu (B.Y.); dl907@njms.rutgers.edu (D.L.); zj89@njms.rutgers.edu (Z.J.); wangq3@njms.rutgers.edu (Q.W.); aisnersc@njms.rutgers.edu (S.A.); 2 Genoptix Medical Laboratory, 2110 Rutherford Road, Carlsbad, CA 92008, USA; dlyle@genoptix.com; 3 The Genomics Center, Rutgers New Jersey Medical School, Newark, NJ 07103, USA; 4 College of Medicine, Drexel University, Philadelphia, PA 19129, USA; aj594@drexel.edu

**Keywords:** Hispanic, Puerto Rico, NSCLC, lung cancer, gene alteration, mutation

## Abstract

**Simple Summary:**

We have analyzed the molecular genetic profiles of Hispanic non-small cell lung cancer (NSCLC) patients from Puerto Rico. In addition to the general characteristics, especially on *EGFR* mutations, we have also reported some novel findings on the incidences of *KRAS* mutation subgroups, other driver gene alterations, and passenger gene alterations, as well as *KRAS/TP53* and *KRAS/STK11* co-mutations. Moreover, our study has identified the *FGFR2-TACC2* translocation in this population.

**Abstract:**

Targeted therapy has changed the paradigm of advanced NSCLC management by improving the survival rate of patients carrying actionable gene alterations using specific inhibitors. The epidemiologic features of these alterations vary among races. Understanding the racial differences benefits drug development, clinical trial design, and health resource allocation. Compared to Caucasian and Asian populations, current knowledge on Hispanic patients is less and no data of Hispanic patients from Puerto Rico have been reported. We retrieved and analyzed the demographic, clinical, and molecular data of Hispanic NSCLC patients from Puerto Rico with molecular tests performed in the Genoptix Medical Laboratory in Carlsbad, CA, USA between 2011 and 2018. The majority of the NSCLC patients in our study had either adenocarcinoma (75.4%) or squamous cell carcinoma (15.1%). The incidence of *EGFR* mutations was 24%. They were more common in female and younger patients (<60 years). The deletion of Exon 19 and Exon 21 L858R comprised 55.1% and 31.0% of all *EGFR* mutations, respectively. The frequency of the T790M mutation was lower compared to that of Hispanic patients reported in the literature (0.5% vs. 2.1%). In addition, 18.7% of the patients were positive for *KRAS* mutations, which was at the high end of that reported in Hispanic patients. Other driver gene alterations, *ALK*, *MET*, *RET*, *ROS1*, *KRAS*, *ERBB2*, etc., demonstrated similar incidences, as well as gender and age distributions to those previously reported. The *KRAS/TP53* and *KRAS/STK11* co-mutations were of very low frequencies (3.6%), which could potentially affect the responsiveness to PD1/PD-L1 immunotherapy. Our study demonstrated that the prevalence of NSCLC gene alterations in Hispanic patients from Puerto Rico was comparable to the reported average prevalence in Latin American countries, supporting the intermediate NSCLC gene alteration rate of Hispanic patients between Asian and Caucasian patients. Novel information of the frequencies of *KRAS* mutation subtypes, driver gene alterations in *ROS1*, *BRAF*, and *ERBB2*, and passenger gene alterations including a rare case with the *FGFR2-TACC2* translocation in Hispanic NSCLC patients from Puerto Rico were also described.

## 1. Introduction

Cancer driver genes refer to the genes whose alterations increase cell proliferation or survival, leading to clonal expansion and tumor growth. *KRAS* is the first driver gene identified in lung cancer patients. It is also the most commonly mutated gene, and found in approximately 22% of the lung cancer patients in western countries [1]. To date, the driver genes identified in lung cancers include *KRAS*, *EGFR*, *ALK*, *MET*, *BRAF*, *ROS1*, *RET*, *ERBB2*, and *NTRK*, most of which contain actionable alterations except *KRAS*. The passenger genes, such as *TP53*, *AKT1*, *PIK3CA*, *MAP2K1*, *STKII*, *KEAP1*, etc. are not involved in the disease initiation, but some have a therapeutic or prognostic value. The identification of genetic alterations and specific inhibitors to these alterations has shifted the management of advanced NSCLC from surgical resection and chemotherapy to targeted therapy. Nowadays, molecular studies detecting these genetic alterations are a routine work-up in lung cancer management. These studies provide information not only for treatment, but also for prognosis and drug resistance.

The racial disparity of gene alterations in lung cancer is well known. For example, the incidence of *EGFR* mutations is higher in Asian patients (~40%), but lower in Caucasian patients (~11%) [2,3]. Studies on the epidemiologic features of lung cancer gene alterations in Hispanic patients are relatively less and of small size except the two conducted by Arrieta et al. [4,5,6,7,8,9,10] in 2011 and 2015. The reported *EGFR* mutation rate in Hispanic patients was between Asian and Caucasian patients, widely ranging from 13% to 37.3%. Hispanic patients had *KRAS* (7–20%) and *ALK* (4.2–10.5%) alteration rates comparable to Asian patients, but lower than Caucasian patients [6]. When breaking down the incidences of the alterations based on countries/regions, we can see a considerable variation though all the patients identified themselves as Hispanics. For example, the *EGFR* mutation rate is 11% in patients from Argentina, but 67% in patients from Peru. The heterogeneity of the Hispanic population is one of the reasons for this large variation. Hence, studying the epidemiologic features of lung cancer gene alterations in Hispanic patients according to the countries or regions they reside in could probably provide more valuable information than viewing them as a pure ethnic group. In this study, we analyzed the molecular genetic profiles of Hispanic NSCLC patients from Puerto Rico, presented the epidemiologic features of more than 20 genes, which expanded the current information on lung cancer gene alterations in Hispanic patients. In addition to the general characteristics, we also reported some novel findings on the incidences of *KRAS* mutation subgroups and passenger gene mutations, as well as *KRAS/TP53* and *KRAS/STKII* co-mutations, which were reported to be associated with an adverse prognosis of NSCLC [11,12,13,14].

## 2. Results

### 2.1. Patient Demographics and Clinical Diagnosis

The age of the patients ranges from 35 to 95 years, with a mean age of 69 years and a median age of 70 years. Moreover, 52.7% (501/951) are male patients and 47.3% (450/951) are female patients. Of these patients, 80.3% are equal to or older than 60 years. Furthermore, 75.4% (717/951) of the patients were diagnosed with adenocarcinoma, 15.1% (144/951) of them with squamous cell carcinoma, and 6.9% (90/951) of them with other types of NSCLC (Table 1).

### 2.2. EGFR Mutation

The *EGFR* status was available in 82% (780/951) of the patients. *EGFR* mutation(s) was identified in 24% (187/780) of them (Table 2), with Exon 19 deletion (55.1%, 103/187) being the most frequent one, followed by Exon 21 L858R (31.0%, 58/187), Exon 20 insertion (4.8%, 9/187), Exon 21 L861Q (2.7%, 5/187), Exon 18 G719S (2.7%, 5/187), and Exon 20 S768I (1.6%, 3/187) (Figure S1). We compared our data with the largest NSCLC mutation study in the Hispanic/Latino population [5,7] available so far (Table 2). The overall *EGFR* mutation rate of our patients is comparable to the reported 26.0% (1491/5738). The Exon 19 deletion had a higher rate in our patient population (55.1% vs. 47.1%, *p* = 0.039). The frequency of Exon 21 L858R was similar to that as previously reported (31.0% vs. 37.3%, *p* = 0.09). The combined percentage (86.1%) of Exon 19 deletion and Exon 21 L858R was higher in our group of patients, but not significantly different from the reported 84.4% among all the EGFR mutations [5]. Exon 20 S768I had a similar frequency to that in the literature (1.6% vs. 3.1%, *p* > 0.05) [7]. The EGFR T790M mutation, which confers resistance to the 1st and 2nd generation tyrosine kinase inhibitors (TKIs), showed a lower rate in our study (4/780, 0.5%) than that reported in earlier studies (85/5738, 1.4%) (*p* < 0.05) [5]. *EGFR* mutations were detected more often in younger and female patients or patients with adenocarcinoma [2]. Our patients from Puerto Rico demonstrated a similar gender, age, and histologic predilections. Moreover, the female patients having *EGFR* mutations were 33.2% (124/373) and male patients were 15.5% (63/407) (*p* < 0.05). The median age of patients with positive *EGFR* mutation was 68 years. In patients younger than 60 years old, the mutation rate was 30.1% (46/153), higher than the 19.5% (80/410) found in patients older than or equal to 60 years (*p* < 0.05) (Table 3). Furthermore, the patients with adenocarcinoma having *EGFR* mutations were 28.2% (169/431), whereas only 5.4% of the patients with squamous cell carcinoma carried *EGFR* mutations (Table S1). The adenocarcinoma rate is significantly higher than that of squamous cell carcinoma (*p* < 0.05).

### 2.3. KRAS Mutation

In our study group, the *KRAS* mutation rate is 18.7% (77/412), significantly higher compared to the reported frequency (14.0%) in Hispanic patients in Arietta’s paper [5] (Table 2). In contrast to the *EGFR* mutations, *KRAS* mutations occurred more often in older patients than in younger patients (29.9% vs. 13.5%, *p* < 0.05). The *KRAS* mutation rate was slightly higher in male patients, with a rate of 21% vs. 16.1% in female patients. However, this difference was not statistically significant (Table 3). A strict mutual exclusion of *EGFR* mutation and *KRAS* mutation was reported previously, but more recent studies have shown an overlap between *KRAS* and *EGFR* in a small number of cases [16,21]. Nine *KRAS* mutations have been described in the literature, with mutations in codon 12 being the most common ones. Some clinical studies demonstrated that G12V and G12C mutations were associated with poor prognosis [13,14]. In addition, codon 12 mutations were detected in 97.4% (75/77) of our patients, and the frequencies of particular codon 12 mutations were as follows: G12C (40.3%), G12V (18.2%), G12D (23.4%), and G12A (6.5%) (Table 4). The co-existence of *KRAS* mutations with secondary mutations has been reported to have some distinctive clinical features, e.g., *KRAS* and *STK11* co-mutations are associated with poor overall survival, and patients with concurrent *KRAS* and *p53* mutations are more sensitive to PD1 and PD-L1 immunotherapy [11,12]. In our patients from Puerto Rico, the co-mutations of *KRAS/p53* or *KRAS/STK11* were 3.6% (2/55) for each combination (Table 4).

### 2.4. ALK Rearrangement

The *ALK* rearrangement is a targetable genetic alteration in NSCLC with a low incidence. The reported *ALK* rearrangement rates in Caucasian, Asian, and Hispanic patients are 1–3%, 2.3–6.7%, and 4.2–10.5%, respectively [8]. In our study, the frequency of *ALK* rearrangement was 3.9% as detected by FISH (28/710) (Table 2). This is within the range of the *ALK* rearrangement rate of Asian patients. The *ALK* rearrangement tends to be present in younger patients, with a median age of 52 years [22]. In our study cohort, the median age of patients with a positive *ALK* rearrangement was 66.5 years, but it occurred more frequently in relatively young patients, with a rearrangement rate of 7.1% (9/127) in patients younger than 60 years and 3.3% (19/583) in patients older than 60 years (*p* < 0.05) (Table 3). Though the incidence of *ALK* rearrangement is reported to have a male predilection [22], we did not see this gender difference in our study (Table 3).

### 2.5. MET Amplification and Mutation

The *MET* gene amplification, mutation, rearrangement, and protein overexpression all lead to an elevated MET protein kinase activity and tumor growth [23]. A secondary *MET* amplification also plays a role in acquired TKI resistance [24]. The *MET* gene amplification is now used as a biomarker to predict the responsiveness to MET inhibitors. In TKI-naïve NSCLC, the incidence of *MET* amplification ranges from 2–5%, and reaches 5–20% in NSCLC with resistance to the 1st or 2nd generation of TKIs [2,16,17,18]. An increased *MET* amplification (≥5 GCN) is an adverse prognostic factor in surgically resected NSCLC [25]. We have tested 172 patients for *MET* amplification by FISH, 10.5% (18/172) of them had increased *MET* gene copy numbers (Table 3). The median age of *MET* amplification patients was 66 years. No age or gender predilection is observed. In the 69 patients tested with NGS results (different from the 172 patients above), five patients carried *MET* mutations, but none of the mutations have a definite clinical significance (Table S2). The bioinformatics analysis revealed that these mutations have no potential to lead the *MET* Exon 14 skipping.

### 2.6. Other Driver Gene Alterations

In our study, 2.2% of the patients had the *ROS1* positive rearrangement (7/322), similar to the reported 0.7–3.4% [15]. In addition, 2.1% of the patients had the *RET* positive rearrangement (4/190), similar to the reported 1.0–3.0% [16]. A total of 94 patients had *BRAF* tested by NGS and four of them were positive (4.3%), which is also comparable to the reported 1–5% in non-ethnic based studies [19]. *ERBB2* was detected by NGS with four positives (6%, 4/69), similar to the documented 2–5% in lung adenocarcinoma (Table 2) [20].

### 2.7. Overlaps of Driver Gene Alterations

Recent publications have reported overlaps of driver mutations though at a very low rate [26,27], and our data supported these observations. Among the driver genes, *BRAF* is the only one showing no overlap with others. We observed two cases with overlapping *KRAS* and *EGFR* mutations, both co-existed with Exon 19 deletion. *ALK*, *ROS1*, *RET*, and *MET* each coexist with *EGFR* in a few cases, *KRAS* coexisted with *MET* in two patients, and *ROS1* and *RET* were both positive in one patient (Figure S2).

### 2.8. Passenger Gene Alterations

Passenger gene alterations were tested in 55 patients by NGS (Table S3). *TP53* had the highest mutation rate, 54.55% (30/55). The incidences of mutations in other genes, such as *STK11*, *IGF1*, *FGFR*, etc., ranged from 0% to 11.24% (Table 5). A very interesting finding is that one of the two cases with *FGFR2* gene alterations harbored the *FGFR2-TACC2* translocation. The patient was an 85-year-old female with a mucinous adenocarcinoma of the lung.

## 3. Discussion

*EGFR* mutations in NSCLC have a strong racial disparity: More common in Asian patients (40–51.4%) than Caucasian patients (9.8–11%) [2,3]. The data on the Hispanic population vary over a wide range. Some of the studies demonstrated that the incidences are between Asian and Caucasian patients, ranging from 24.6–35.3% [5,7,8]. Others showed lower *EGFR* frequencies (13–18%) and no difference from that seen in Caucasian patients [4,8,9,10]. The *EGFR* mutation rate of patients from Puerto Rico in our study was 24.0%, consistent with the intermediate *EGFR* mutation rate in Hispanic NSCLC patients. The Exon 19 deletion and Exon 21 L858R mutation are the two most common *EGFR* mutations, comprising 85–90% of all the *EGFR* mutations, with Exon 19 deletion slightly more common than Exon 21 L858R [2]. These two mutations are associated with a better prognosis regardless of the treatment [2]. The frequency of Exon 19 deletion in our study was higher than the reported incidence in Arrieta’s paper (55.1% vs. 47.1%) (*p* < 0.05), and the Exon 21 L858R mutation rate was relatively lower but with no significant difference (31.0% vs. 37.3%, *p* > 0.05). The combined incidence of Exon 19 deletion and Exon 21 L858R mutation (86.1%) was comparable to that published in the literature. There is no difference between the Exon 19 deletion and Exon 21 L858R mutation regarding their responsiveness to therapy or disease prognosis. Therefore, the above difference may not be clinically significant. The *EGFR* T790M mutation is one of the mechanisms for the tyrosine kinase inhibitor (TKI) resistance. It can either occur as a primary mutation or as an acquired mutation following the TKI treatment. The T790M mutation can be as high as ~50% in patients treated with TKIs, whereas the treatment-naive mutations are reported to be less than 1% [28,29]. This mutation almost always coexists with either the Exon 19 deletion or the L858R mutation. The Exon 19 deletions are more commonly present with the acquired T790M but the L858R is more likely with the primary T790M [28]. In our study, out of 780 patients with positive *EGFR* mutations, only four of them (0.4%) have a concomitant T790M, which is within the range of the reported incidence of primary T790M. All the four cases had the Exon 19 deletion. Due to the lack of treatment information, we were not able to determine whether the T790M mutations were primary or acquired. The best interpretation of this low occurrence could be the low prevalence of TKI use in our study population.

Globally, *KRAS* mutations are the most common mutations in NSCLC and considered a negative prognostic factor [30]. Asian patients have a lower *KRAS* mutation rate (8–15%) compared to patients from western countries (18–28.1%) [13,31,32]. The frequencies of *KRAS* mutations in Hispanic/Latino patients are reported between 7–20, comparable to that in the Asian population [5,6,8,33,34]. In our study cohort the *KRAS* mutation rate was 18.7%. This mutation rate falls into the reported range of Hispanic patients but is significantly higher than that reported in the Arietta’s paper [5]. Most *KRAS* mutations were located at codons 12, 13, and 61. Some mutation subtypes are associated with an adverse prognosis. G12V and G12C portend an inferior overall survival compared to the G12A and G12D mutations [13,14]. In our study, 97.4% of the patients had codon 12 mutations (75/77), and the frequencies of individual mutations were as follows: G12C (40.3%), G12V (18.2%), G12D (23.4%), and G12A (6.5%). G12V and G12C combined constitute 58.4% (45/77) of all *KRAS* mutations, and G12A and G12D account for 29.9% (23/77). These numbers were similar to the reported values: G12C (39%), G12V (18–21%), G12D (17–18%), and G12A (10.8%) [13,14].

Though the development of specific inhibitors to *KRAS* or its downstream signaling molecules has been a difficult journey without success so far, recent studies show that the *KRAS* co-mutation status in NSCLC is associated with its responsiveness to the PD-1/PD-L1 immunotherapy, as well as the disease prognosis [34]. Lung adenocarcinomas with concurrent *KRAS* and *TP53* mutations are more responsive to PD1/PD-L1 inhibitors, whereas tumors with *KRAS* and *STKII* co-mutations exhibit resistance to those inhibitors [11,12]. Patients with *KRAS* and *STKII* co-mutations or with the *STKII* mutation alone also have poor overall survival compared to patients with no concurrent mutations, wild type *KRAS*, or wild type *STKII* [12]. Our study cohort included 55 patients with available *KRAS*, *TP53*, and *STKII* mutation statuses available for analysis. Our current observation is the first study reporting data of concurrent *KRAS/TP53* and *KRAS/STKII* mutations in the Hispanic population. Surprisingly, these concurrent mutations in Hispanic NSCLC from Puerto Rico were very rare; only two cases for each combination (3.6%), compared to the published data (52% and 18%, respectively) [14]. Therefore, in Puerto Rican patients, the *KRAS* co-mutation status may not be a proper biomarker to estimate the responsiveness to immunotherapy. Moreover, the rarity of these co-mutations in Puerto Rican patients makes them an unfavorable population for studying these co-mutations. The low incidences cannot be explained by each individual frequency of *KRAS*, *TP53*, and *STK11* mutations. The *KRAS* mutation frequency was relatively lower (18.7 vs. 27%) in our study [14], but the frequencies of *STK11* and *TP53* mutations were similar to the reported values (11% vs. 8–18% for *STK11*; 55% vs. 46% for *TP53*) [16,35,36].

**Figure 1:**
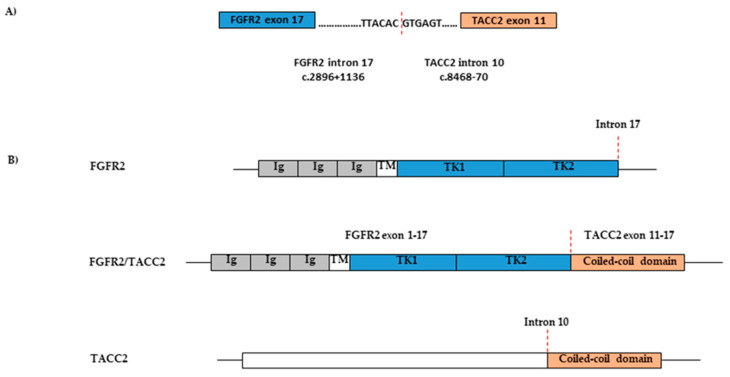
Predicted protein structure of *FGFR2-TACC2* fusion. (**A**) Sequence of the region fused between *FGFR2* and *TACC2* in genome. (**B**) Schematic of *FGFR2-TACC2* fusion involving exons 1 to 17 of *FGFR2* and exons 11 to 17 of *TACC2*. Red dashed lines indicate breakpoints. *FGFR2*, fibroblast growth factor receptor 2; *TACC2*, transforming acidic coiled-coil-containing protein 2; Ig, disulfide linked immunoglobin-like domains; TM, transmembrane domain; TK, intracellular tyrosine kinase domain.

The chromosomal translocation joining in-frame members of the fibroblast growth factor receptor-transforming acidic coiled-coil gene families (*FGFR-TACC* gene fusions) were first identified in a human glioblastoma multiforme [37]. The most common fusion type is *FGFR3-TACC3*, which has been discovered in many cancer types including lung cancer. The fusion between *FGFR* and *TACC* genes results in a constitutively activated kinase, which induces mitotic and chromosomal segregation defects, subsequently triggering aneuploidy. Clinical data showed promising effects of FGFR inhibitors in malignant tumors harboring *FGFR-TACC* fusions [37,38,39]. The rearrangement involving *FGFR2-TACC2* had never been reported in lung cancer before. Our study identified a novel actionable rearrangement involving *FGFR2-TACC2* in one of 55 patients tested by NGS. Figure 1 diagrams the predicted protein structure of the *FGFR2-TACC2* fusion from this patient. The patient presented with mucinous adenocarcinoma of the lung. Interestingly, there is another report of a *FGFR2-TACC2* translocation identified in a mucinous stomach adenocarcinoma in The Cancer Genome Atlas (TCGA) PanCancer studies (TCGA-BR-8080-01). The findings warrant further investigation of the *FGFR2-TACC2* translocation in mucinous adenocarcinoma from different origins.

To the best of our knowledge, our study cohort is the first study on the frequencies of *KRAS* mutation subtypes, driver gene *ROS1* rearrangement, drive gene *BRAF* and *ERBB2* mutations, and passenger gene alterations including the *FGFR2-TACC2* translocation in Hispanic NSCLC patients.

## 4. Materials and Methods

### 4.1. Patients

We conducted a retrospective study of lung cancer gene alterations in Hispanic NSCLC patients from Puerto Rico, who had lung cancer molecular tests performed between 2011 and 2018, in the Genoptix Medical Laboratory in Carlsbad, CA, USA. The diagnosis of NSCLC is based on the information on the requisition forms provided by the requesting clinicians. The specimens were submitted to the Genoptix Medical Laboratory randomly from twenty medical centers or doctor offices across the whole territory of Puerto Rico. The IRB approval (ID: 6173) to perform a retrospective chart review to collect and analyze clinical data, including laboratory, FISH, mutational, demographic, and pathological data, was issued by the Sterling IRB ethic committee on 11 December 2017.

### 4.2. Next Generation Sequencing

The Lung NGS panel (amplicon-based, targeted) of 25 genes was conducted on an Illumina MidiSeq instrument using genomic DNA isolated from unstained FFPE slides. The targeted genes include *AKT1*, *ALK*, *ARAF*, *BCL2*, *BRAF*, *DDR2*, *EGFR*, *ERBB2*, *FGFR1*, *FGFR2*, *FGFR3*, *HRAS*, *IGF1*, *KRAS*, *MAP2K*, *MDM2*, *MET*, *NRAS*, *PDGFRA*, *PIK3CA*, *PTEN*, *RET*, *ROS1*, *STK11*, *TP53*. The alterations within each of those genes were analyzed through the proprietary bioinformatic software and interpreted in conjunction with reference databases such as COSMIC and dbSNP. Quality control metrics included a minimum input of 20 ng, with an optimal input of 100 ng of genomic DNA and average mean sequencing depth of 500× coverage. The limits of detection (LOD) were 5% for SNV, 10% for Indels, ≥6 copies for gene amplifications, and ≤0.3 copies for homozygous gene deletions. Insertions greater than 15 nucleotides and deletions greater than 52 nucleotides may not be detected. Benign sequence variants were not reported.

### 4.3. Multiplex PCR

Mutations of *EGFR*, *KRAS*, and *BRAF* were detected using multiplex PCR, including Exon 19 deletion, Exon 20 insertion, L858R, S786I, G719X, L861Q, and T790M for *EGFR*, mutations in codon 12, 13, and 61 for *KRAS*, and V600E for *BRAF*.

### 4.4. FISH

Gene rearrangements of *ALK*, *ROS1*, and *RET* were detected on unstained FFPE slides by a fluorescent break apart (BA) DNA probes and gene amplification of *MET* was detected on unstained FFPE slides by fluorescent *MET* and CEP7 DNA probes. Fifty cells for *ALK* or sixty cells for *MET*, *ROS1*, and *RET* were analyzed for each case. Accordingly, 15% or more of the tumor cells showing split signals of the fluorescent probes was considered positive (Figure S3). Based on the *MET*-CN and *MET*/CEP7 ratio, the patients’ *MET* results were classified as an *MET*-amplification: *MET*/CEP7 ≥ 2 or *MET*-CN ≥ 5. Fusion partners could not be identified in these FISH assays with break apart DNA probes.

### 4.5. Statistical Analysis

The categorical variables in this study were analyzed using the χ^2^ test and *p* < 0.05 was considered statistically significant.

## 5. Conclusions

In summary, targeted therapy is the trend of medicine. Understanding the epidemiologic features of the genetic alterations in NSCLC in different racial groups improves the efficacy and predictability of the treatment. Our data indicate that Hispanic patients from Puerto Rico showed an intermediate rate of *EGFR* mutations and *ALK* rearrangements, similar to that in most Latin American countries. The *KRAS* mutation rate was at the high end of the reported frequencies in the Hispanic patients, and close to the rate of Caucasian patients. The frequencies of *KRAS* mutation subtypes, driver gene alterations in *ROS1*, *BRAF*, and *ERBB2*, and passenger gene alterations including a rare case with the *FGFR2-TACC2* translocation are novel information in this racial group, which need confirmation by other independent studies.

## Figures and Tables

**Table 1:** Demographic and pathologic features.

Variables	Number (%)
Gender	
Male	501 (52.7%)
Female	450 (47.3%)
Age (years)	
Median (range)	70 (35–95)
Mean ± SD	69 ± 10.43
<60 years	187 (19.7%)
≥60 years	764 (80.3%)
Diagnosis	
Adenocarcinoma	717 (75.4%)
Squamous cell carcinoma	144 (15.1%)
Others	90 (9.5%)

**Table 2:** Alteration incidences of the driver genes.

Genes	Incidence	Reported Incidence	*p*-Value
*EGFR*	24.0% (187/780)	26.0% (1491/5738) [5]	*p* = 0.2282
Exon 19 deletion	55.1% (103/187)	47.1% (702/1491) [5]	*p* = 0.0391 †
Exon 21 L858R	31.0% (58/187)	37.3% (556/1491) [5]	*p* = 0. 0931
Exon 20 S768I	1.6% (3/187)	3.1% (12/382) [7]	*p* = 0.2824
T790M	0.5% (4/780)	1.4% (85/5738) [5]	*p* = 0.0268 †
Others	10.2% (19/187)	N/A	N/A
*KRAS*	18.7% (77/411)	14.0% (190/1355) [5]	*p* = 0.0195 †
*ALK*	3.9% (28/710)	4.2–10.5% [8]	N/A
*ROS1*	2.2% (7/322)	0.7–3.4% [15]	N/A
*RET*	2.1% (4/190)	1.0–3.0% [16]	N/A
*MET* (Amplification)	10.2% (18/172)	2–20% [2,16,17,18]	N/A
*BRAF*	4.3% (4/94)	1–5% [19]	N/A
*ERBB2*	5.8% (4/69)	2–5% [20]	N/A

† *p* < 0.05.

**Table 3:** Gene alterations in relation to gender and age.

Genes	Gender			Age			
	Male	Female	*p*-Value	Median (Years)	<60 Years	≥60 Years	*p*-Value
*EGFR*	15.5%	33.2%	*p* < 0.00001 †	68	30.1%	19.5%	*p* = 0.0075 †
	(63/407)	(124/373)			(46/153)	(80/410)	
*KRAS*	21.0%	16.1%	*p* = 0.1991	72	13.5%	29.9%	*p* = 0.0052 †
	(46/219)	(31/193)			(10/74)	(67/224)	
*ALK*	3.4%	4.6%	*p* = 0.4335	66.5	7.1%	3.3%	*p* = 0.0446 †
	(13/381)	(15/329)			(9/127)	(19/583)	
*MET*	7.4%	14.3%	*p* = 0.1406	66	20.0%	8.8%	*p* = 0.0920
	(7/95)	(11/77)			(5/25)	(13/147)	

† *p* < 0.05.

**Table 4:** KRAS mutation subtypes and co-mutations.

Gene Mutations	Percentage
*KRAS*	
Condon 12	97.4% (75/77)
G12C	40.3% (31/77)
G12V	18.2% (14/77)
G12D	23.4% (18/77)
G12A	6.5% (5/77)
*KRAS* + *TP53*	3.6% (2/55)
*KRAS* + *STK11*	3.6% (2/55)

**Table 5:** Frequencies of passenger gene alterations.

Genes	Positive (n)	Tested (n)	Percentage (%)
*TP53*	30	55	54.6
*STK11*	6	55	10.9
*FGFR1*	6	55	10.9
*PIK3CA*	5	55	9.1
*IGF1*	5	55	9.1
*DDR2*	4	55	7.3
*MDM2*	3	55	5.5
*PTEN*	2	55	3.6
*FGFR2*	2 ^#^	55	3.6
*FGFR3*	2	55	3.6
*HRAS*	1	55	1.8
*PDGFRA*	1	55	1.8
*BCL2*	1	55	1.8
*AKT1*	0	55	0
*ARAF*	0	55	0
*MAP2K1*	0	55	0

^#^: One case with the *FGFR2-TACC2* fusion.

## Supplementary Materials

The following are available online at https://www.mdpi.com/2072-6694/12/12/3492/s1. Table S1: *EGFR* mutations in different histologic types; Table S2: *MET* mutations detected by NGS; Table S3: Clinical data of 55 patients with passenger gene mutations; Figure S1: *EGFR* mutation composition; Figure S2: Overlap of driver gene alterations; Figure S3: Representative FISH figures.
