# Supplementary material for: Epidemiologic Features of NSCLC Gene Alterations in Hispanic Patients from Puerto Rico
Source: Cancers (Basel). 2020 Nov 24;12(12):3492. doi: 10.3390/cancers12123492 (PMC7761356; doi:10.3390/cancers12123492)
Supplement: Supplementary file 1 [file cancers-12-03492-s001.pdf]

cancers

Supplementary Materials: Epidemiologic Features of NSCLC Gene Alterations in Hispanic Patients from Puerto Rico

Ruifang Zheng, Zhiwei Yin, Albert Alhatem, Derek Lyle, Bei You, Andrew S. Jiang, Dongfang Liu, Zsolt Jobbagy, Qing Wang, Seena Aisner and Jie-Gen Jiang

Table S1. *EGFR* mutations in different histologic types.

Histologic Types	*EGFR* Positive	*EGFR* Negative	%
Adenocarcinoma	169	431	28.2%
Squamous cell carcinoma	6	105	5.4%
Other types	12	59	16.9%

Table S2. *MET* mutations detected by NGS.

Patient#	Age (years)/Gender	Mutation site	Mutation type
1	76/F	c.3039G>C; p.M1013I	MISSENSE
2	65/M	c.1771C>T; p.R591W	MISSENSE
3	57/F	c.1039G>A; p.A347T	MISSENSE
4	68/F	c.2201G>A; p.S734N	MISSENSE
5	76/M	c.1393-2A>C;	SNP

Table S3. Clinical data of 55 patients with passenger gene mutations.

Age (years)	Gender	Histopathology diagnosis
65	F	Metastatic adenocarcinoma of lung primary
78	M	Squamous carcinoma of the lung
76	M	Adenocarcinoma of the lung
68	F	Adenocarcinoma of the lung, well differentiated
65	M	Squamous cell carcinoma of the lung
67	F	Adenocarcinoma of the lung with papillary features
80	F	Adenocarcinoma of the lung
75	M	Mucinous adenocarcinoma of the lung
65	F	Squamous cell carcinoma of the lung
72	F	Adenocarcinoma of the lung
57	F	Metastatic adenocarcinoma of lung primary
77	F	Metastatic adenocarcinoma of lung primary
60	M	Metastatic adenocarcinoma of lung primary
65	M	Metastatic adenocarcinoma of lung primary, moderately differentiated
72	F	Adenocarcinoma of the lung
67	F	Non-small cell type carcinoma of the lung, adenocarcinoma subtype.
54	M	Metastatic large cell carcinoma of lung primary, with features of adenocarcinoma.
72	M	Squamous cell carcinoma of the lung
84	F	Adenocarcinoma of the lung, moderately differentiated
85	F	Mucinous adenocarcinoma of the lung with *FGFR2-TACC2* translocation
81	M	Adenocarcinoma of the lung, moderately differentiated

63	F	Adenocarcinoma of the lung
58	F	Adenocarcinoma of the lung, well-differentiated
76	M	Adenocarcinoma of the lung, moderately differentiated
64	M	Metastatic adenocarcinoma of lung primary.
79	M	Metastatic adenocarcinoma of lung primary
84	F	Adenocarcinoma of lung primary.
68	F	Adenocarcinoma of the lung
72	M	Adenocarcinoma of the lung, moderately differentiated
46	M	Adenocarcinoma of bronchogenic origin
70	M	Squamous carcinoma of the lung, moderately differentiated
83	M	Non-small cell lung carcinoma, adenocarcinoma subtype.
64	M	Squamous cell carcinoma of the lung, moderately differentiated
51	F	Adenocarcinoma of the lung, well differentiated
31	F	Adeno-squamous cell carcinoma of the lung
39	F	Adenocarcinoma of lung origin
79	F	Adenocarcinoma of the lung
78	M	Adenocarcinoma of the lung
46	M	Carcinosarcoma of the lung
61	M	Adenocarcinoma of the lung, poorly differentiated
76	M	Non-small cell lung carcinoma, favor adenocarcinoma.
50	F	Non-small cell lung carcinoma, favoring adenocarcinoma (bronchio-alveolar).
66	F	Adenocarcinoma of the lung
68	F	Mucinous adenocarcinoma of lung origin
70	F	Adenocarcinoma of the lung
86	F	Non-small cell lung carcinoma, favor adenocarcinoma.
75	M	Non-small cell lung carcinoma, favor squamous cell carcinoma.
69	F	Non-small cell carcinoma, favor adenocarcinoma.
60	F	Neuroendocrine carcinoma of the lung, well differentiated
67	F	Non-small cell lung carcinoma, poorly differentiated
71	M	Metastatic adenocarcinoma of lung primary.
50	F	Adenocarcinoma of the lung
85	M	Squamous cell carcinoma of the lung
66	F	Adenocarcinoma of the lung, well-differentiated
55	M	Metastatic adenocarcinoma of lung primary.

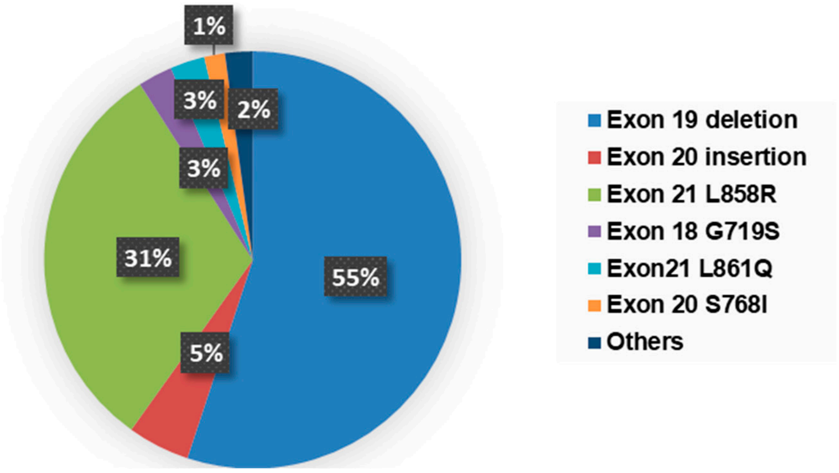

Figure S1: *EGFR* mutation composition

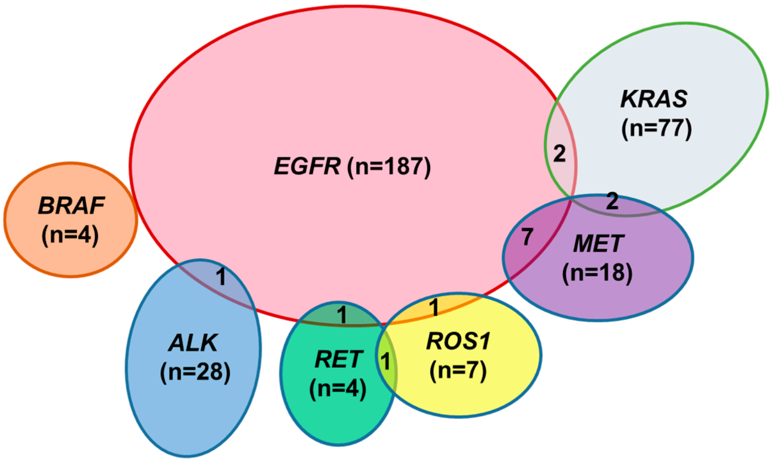

Figure S2. Overlap of driver gene alterations.

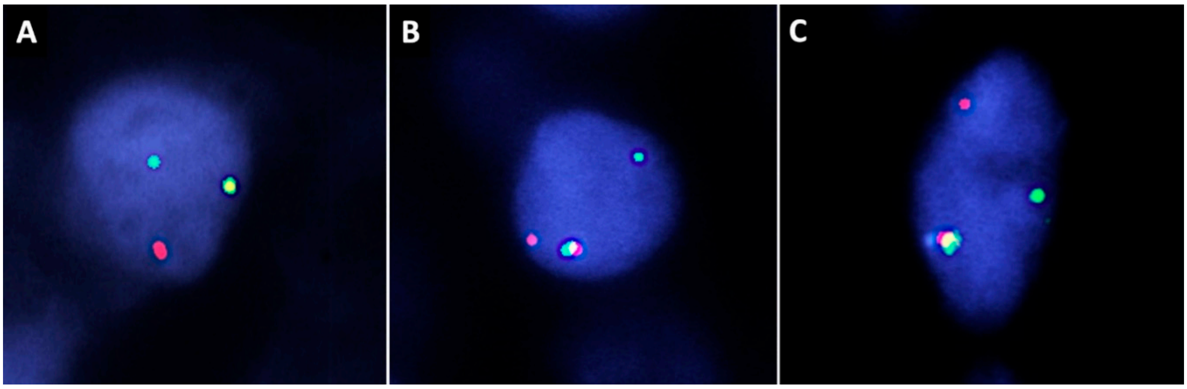

Figure S3. Representative FISH figures. (A) *ALK* rearrangement-one red, one green, and one yellow with the *ALK* BA probe; (B) *ROS1* rearrangement-one red, one green, and one yellow with the *ROS1* BA probe; (C) *RET* rearrangement-one red, one green, and one yellow with the *RET* BA probe; Separate red and green signals indicate rearrangements. The yellow signals indicate intact genes with no rearrangements.
